# Pharmacokinetic characterization of three novel 4-mg nicotine lozenges 

**DOI:** 10.5414/CP203097

**Published:** 2018-01-19

**Authors:** Manpreet Sukhija, Reena Srivastava, Aditya Kaushik

**Affiliations:** GlaxoSmithKline Consumer Healthcare, Gurgaon, Haryana, India; * Manpreet Sukhija and Aditya Kaushik are former employees of GlaxoSmithKline Consumer Healthcare, Haryana, India, and were employed by Glaxo­SmithKline Consumer Healthcare at the time this study was conducted.

**Keywords:** tobacco use cessation products, tobacco, smoking cessation, smoking, nicotine replacement therapy

## Abstract

Objective: Nicotine replacement therapy (NRT) increases the probability of smoking cessation. This study was conducted to determine if three prototype 4-mg nicotine lozenges produced locally in India were bioequivalent to a globally marketed reference product, Nicorette^®^ 4-mg nicotine lozenge. Materials and methods: Healthy adult smokers (N = 39) were treated with three prototype 4-mg nicotine lozenges in comparison with a reference 4-mg lozenge in this single-center, randomized, open-label, single-dose, 4-way crossover study. Pharmacokinetic sampling was obtained to test for bioequivalence using maximal plasma concentration (C_max_) and extent of absorption (AUC_0–t_). Secondarily, AUC_0–∞_, time to maximal plasma concentration (t_max_), half-life (T_1/2_), elimination rate constant (K_el_), and safety of the prototype lozenges versus the reference lozenge were compared. Results: Each prototype 4-mg nicotine lozenge was found to be bioequivalent to the reference 4-mg nicotine lozenge based on the ratio of geometric means and 90% confidence intervals for C_max_, AUC_0–t_, and AUC_0–∞_. Although t_max_ was significantly longer for prototype III, all four lozenges achieved maximum plasma nicotine concentrations at a median of 1.5 hours. The safety profiles of the three prototype 4-mg lozenges did not differ from that of the 4-mg reference product. Conclusion: Each prototype 4-mg nicotine lozenge was bioequivalent to the reference 4-mg nicotine lozenge and was well tolerated. Furthermore, as these bioequivalent prototypes differed in in-vitro dissolution profiles, these data suggest that performance from the in vitro method deployed is not a firm predictor of pharmacokinetic behavior.

## Introduction 

Tobacco use is a leading preventable cause of disease and death. Smoking damages nearly every organ of the body and causes health problems including cardiovascular disease, respiratory disease, cancer, and fertility problems in women [1]. Of the more than 1 billion smokers globally, 80% live in low- and middle-income countries, and ~ 7 million die each year from both direct tobacco use and second-hand smoke [2]. 

In India, the prevalence of smoking and smokeless tobacco use has been estimated at ~ 30% of adults (> 193 million people) [3], and the World Health Organization estimates that ~ 13% of the population smoked in 2010, including 24% of men and 3% of women [4]. According to data from the Global Adult Tobacco Survey India (GATS-India), smoking is more prevalent among Indians who are male, older, less educated, and living in rural areas [5]. 

One recent Gallup survey reported that 74% of current smokers would like to quit [6]. This is not an easy task given that the rapid delivery of nicotine to the brain and ease with which the dose can be titrated via smoking encourages repeated self-administration and promotes addiction [7]. Nicotine replacement therapies (NRTs) are medications designed to assist smokers with quitting by delivering nicotine to the brain to reduce withdrawal symptoms without delivering the toxins produced by cigarette smoking [8]. Clinical guidelines recommend multiple forms of NRT as first-line medications to effect smoking cessation, including lozenge, gum, transdermal patch, inhaler, and nasal spray formulations [9]. Unlike more expensive prescription smoking cessation aids such as bupropion and varenicline, many NRT products are available over the counter, making them more accessible [8]. NRTs were added to the Model List of Essential Medicines by the World Health Organization in 2009 to facilitate their access globally [10]. 

Nicotine lozenges have been shown in a randomized, placebo-controlled trial to more than double the chances of a smoker staying abstinent for a year [11]. Nicotine lozenges are currently available in India as both over-the-counter and prescription products. Among the nicotine lozenges available in India, those produced and marketed globally by GlaxoSmithKline Consumer Healthcare are provided in 2-mg and 4-mg dosage strengths. The GSK affiliate in India (GlaxoSmithKline Asia Private Limited) has developed nicotine lozenges in these strengths to be produced locally. In this study, the pharmacokinetic (PK) parameters of three prototype formulations of 4-mg nicotine lozenges manufactured in India were compared to those of the globally marketed 4-mg nicotine lozenge to determine if the products are bioequivalent. 

## Materials and methods 

### Study design 

This was a single-center, randomized, open-label, single-dose, 4-way crossover study conducted between June 4 and July 5, 2012 in full conformance with the laws and regulations of India and with the requirements specified in the Declaration of Helsinki, the International Council for Harmonisation Guideline for Good Clinical Practice, and other applicable regulations. Before initiation of the study, Independent Ethics Committee-Aditya reviewed and approved the protocol and informed consent form, and regulatory approval for the study was obtained from the Drug Controller General of India (DCGI). The study was registered on ClinicalTrials.gov (NCT01669122). 

### Study subjects 

Subjects were men aged 18 – 45 years who had smoked daily for at least the previous 12 months, routinely smoked their first cigarette within 30 minutes of awakening, and were in otherwise good general health. Subjects agreed to abstain from smoking or use of other tobacco products during the confinement period (at least 36 hours prior to dosing and through the collection of the last PK sample). Potential subjects were excluded if they had unstable physical or psychiatric illness, any disease that could affect the action, absorption, or disposition of the study medication; oral surgery within 4 weeks or dental work/extractions within 2 weeks of dosing; used a prescription, over-the-counter, or herbal medication within 7 days or drugs that induce/inhibit hepatic drug metabolism within 30 days of study medication dosing; or had a positive screen for hepatitis B or C or human immunodeficiency virus. In addition, a history of phenylketonuria; history of allergy or intolerance to any of the study materials; recent history (within 1 year) of drug or alcohol abuse; positive urine drug screen at screening or baseline or alcohol breath test at baseline; participation in another clinical study or receipt of an investigational drug within 30 days of screening; or blood donation or significant blood loss within 3 months of screening were exclusionary. Potential subjects were selected from a database of volunteer smokers. 

### Screening 

Potential subjects who fulfilled inclusion/exclusion criteria were informed about the purpose and conduct of the study, and those who wished to participate provided informed consent. Demographic data, medical history, and vital signs including body mass index were collected from potential subjects. Clinical assessment included physical examination, 12-lead electrocardiogram, chest X-ray, clinical laboratory tests (hematology, serum chemistries, and virology testing), urinalysis, urine drug screening, and assessments of concomitant medication and smoking history. Subjects with clinically significant abnormalities from these assessments were withdrawn. 

### Study sessions 

Study sessions (four total) each consisted of a baseline phase and a treatment phase. At baseline, eligible subjects were confined to the study facility for ~ 36 hours before receiving the study treatment. During each baseline phase, continued eligibility was confirmed with reassessment of medical history, concomitant medications, physical examination, vital signs, and inclusion/exclusion criteria. In addition, hemoglobin level, urine drug screen, and breath alcohol testing were conducted. Exhaled carbon monoxide (CO) measurements were taken upon check-in at baseline and immediately before dose administration. During the treatment phase (lasting ~ 14 hours), exhaled CO measurements were obtained immediately after the last PK sample was obtained and at four randomly chosen times during this phase to verify smoking abstinence, given that smoking was not allowed during study sessions. Subjects with CO values greater than 10 parts-per-million were withdrawn from the study. Subjects were permitted to smoke freely between study sessions, and consecutive sessions were separated by a washout period of at least 48 hours. 

On day –1 of each study session, subjects fasted from food beginning at 10 PM and from fluids beginning at midnight; fasting until 4 hours after dosing was required. Dosing occurred at ~ 8 AM on day 1, and water was allowed at all times except from 1 hour before and 1 hour after dosing. Subjects consumed standard meals provided by the study site, and no alcohol was permitted during confinement. Finally, subjects were asked to refrain from caffeine and not undertake any strenuous physical exercise from 24 hours before dosing until the end of the session. 

### Study treatments 

The three prototype 4-mg nicotine lozenges (prototypes I, II, and III), which differ in their dissolution properties and nicotine-release profiles, were supplied by the New Product Development Department – Wellness and Oral HealthCare, GlaxoSmithKline Asia Private Limited, Gurgaon, India, and were manufactured by Kemwell Biopharm Private, Bangalore, India. An internationally marketed 4-mg nicotine lozenge (Nicorette^®^, GlaxoSmithKline Consumer Healthcare, Warren, NJ, USA) was used as the reference product for PK analysis. 

At each study session, subjects received one of the four study treatments according to a computerized randomization schedule generated by the Biostatistics Department at GlaxoSmithKline Consumer Healthcare; in total, 24 treatment sequences were used. Nicotine lozenges were administered as a single 4-mg dose, and subjects were instructed not to swallow or chew the lozenge; time to complete dissolution of the dose was recorded by investigators. 

### Pharmacokinetic analysis 

Blood samples were drawn from subjects immediately before dosing and at 14 timepoints (5, 10, 15, 30, and 45 minutes and 1, 1.5, 2, 3, 4, 6, 8, 10, and 12 hours) after dosing. Approximately 424.0 mL of blood was collected from each subject over the course of the study. The blood sampling times were chosen to maximize the information available, particularly on nicotine absorption and elimination, taking into account that nicotine has a half-life in the blood of ~ 120 minutes. Samples were collected in vacutainers containing K_2_EDTA anticoagulant, mixed via inversion, and placed in an ice-water bath. Blood samples were centrifuged at 3,000 ± 100 relative centrifugal force for 5 minutes below 10 °C to separate plasma, which was then aliquoted and frozen at –65 ± 10 °C for transfer to the bioanalytical laboratory (Lambda Therapeutic Research Ltd., Gujarat, India). The plasma samples were analyzed for nicotine concentration using a validated liquid chromatography tandem mass spectroscopy (LC-MS/MS) method. The lower limit of quantification for nicotine was 0.987 ng/mL. 

### Safety assessment 

Adverse events (AEs), AE severity, and relationship to study treatment were recorded by investigators. Serious AEs, defined as any AE that resulted in death, was life-threatening, required hospitalization or prolonged an existing hospitalization, resulted in disability/incapacity, or was a congenital anomaly/birth defect, were also recorded. Vital signs and laboratory assessment results were recorded. 

### Statistical analysis 

A total of 40 subjects were to be randomized to ensure that at least 32 subjects completed all four treatment arms of the study; however, no formal sample-size calculation was performed. For purposes of analysis, the intent-to-treat (ITT) population included all subjects who received at least one of the study treatments and provided enough plasma samples to estimate the PK parameters. The per-protocol (PP) population included all subjects without protocol violations and with enough nicotine concentration data to determine the PK parameters. The safety population consisted of all subjects who were dispensed at least one study treatment, regardless of inclusion in the PK analysis. 

Descriptive statistics were used for demographic and baseline data. The primary PK parameters calculated were area under the plasma concentration-vs.-time curve from zero extrapolated to the time of the last quantifiable sample (AUC_0–t_) and maximum plasma nicotine concentration (C_max_). Plasma nicotine concentration data were adjusted to account for measurable pretreatment nicotine levels, and the primary analysis was conducted using baseline-adjusted data. 

A linear mixed-effects model was used to analyze the logarithmically transformed (natural log) primary parameters (AUC_0–t_ and C_max_) using the PROC MIXED procedure in the Statistical Analysis System software suite (SAS Institute Inc., Cary, NC, USA). The model included subjects as a random effect and treatment and study period as fixed effects. The residual variance from the model was used to construct 90% confidence intervals (CIs) for the difference between the prototype and reference treatments. The CIs were then back-transformed to obtain point estimates and CIs for the ratio of the treatment geometric means. The prototype lozenges were determined to be bioequivalent if the 90% CIs for the ratios of the geometric means of AUC_0–t_ and C_max_ fell within the predefined limits of 0.80 and 1.25. 

Secondary PK parameters were calculated including AUC from zero extrapolated to infinity (AUC_0–∞_), time to C_max_ (t_max_), plasma elimination rate constant (K_el_), and plasma half-life (T_1/2_). AUC_0–∞_ was analyzed in the same manner as AUC_0–t_ and C_max_, t_max_ was analyzed using the nonparametric Wilcoxon signed-rank test, and median differences between the prototype and reference formulations were determined with 95% CIs based on the one-sample method by Hodges and Lehmann. K_el_ and T_1/2_ were summarized using descriptive statistics. All AEs and SAEs were summarized by treatment group. 

## Results 

### Subject disposition 

Of 102 subjects screened, 40 were randomized, and 37 completed all four periods of the study. Three subjects withdrew from the study (2 due to protocol violations and 1 who was lost to follow-up). The ITT, PP, and safety populations each comprised 39 subjects. Subject demographics and baseline characteristics are shown in Table 1. All subjects were Asian men aged between 20 and 44 years; none had physical examination abnormalities, exclusionary medical histories, or concomitant medication use. 

### Pharmacokinetic results 

**Figure 1.**
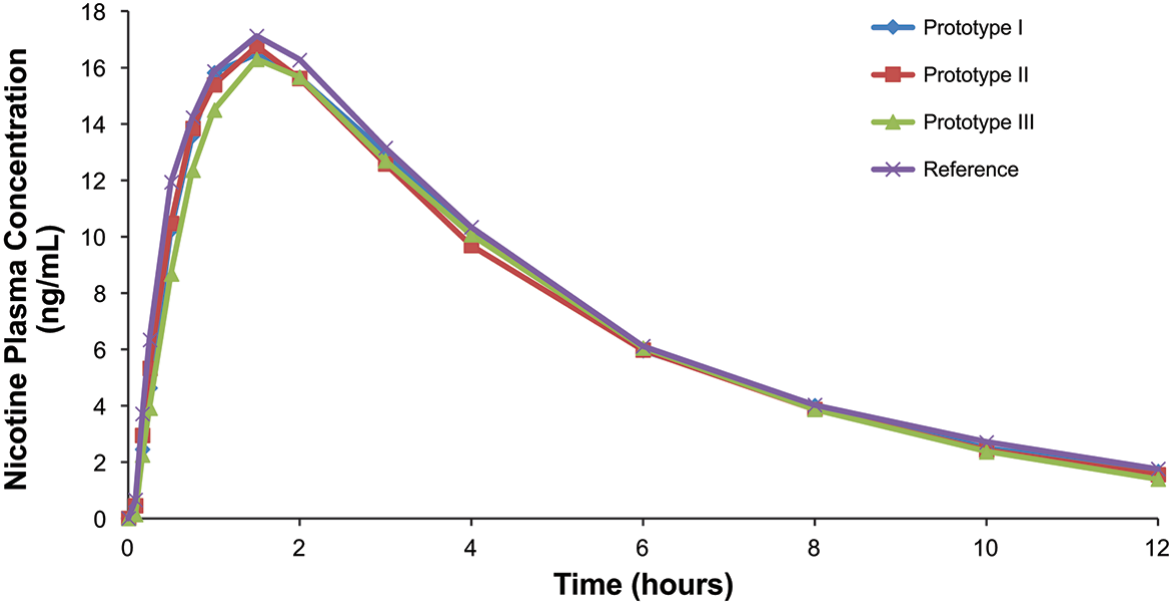
Baseline-adjusted mean plasma nicotine concentration over time.

Baseline-adjusted nicotine plasma PK variables for the three prototype 4-mg nicotine lozenges and the marketed 4-mg nicotine lozenge are provided in Table 2; corresponding baseline-adjusted plasma nicotine-vs.-time curves are provided in Figure 1. In the primary PK comparison of the three prototype lozenges versus the reference lozenge, the ratio of the geometric means (prototype/reference) and 90% CIs for C_max_ and AUC_0–t_ each fell within the accepted bioequivalence limits of 0.80 to 1.25, indicating that each prototype lozenge was bioequivalent to the reference lozenge (Table 3). Evaluation of the secondary PK parameter AUC_0–∞_ revealed that the geometric mean ratio and 90% CI for each of the 3 prototype lozenges also fell within the 0.80 – 1.25 limits, supporting the results of the primary PK analysis (Table 3). Similarly, analyses of unadjusted data also demonstrated the bioequivalence of all 3 prototype lozenges to the reference lozenge in terms of C_max_, AUC_0–t_, and AUC_0–∞_ (data not shown). Based on the baseline-adjusted analyses, t_max_ was significantly greater (p = 0.0063) for prototype lozenge III versus the reference lozenge, whereas no significant differences were observed for prototype lozenges I and II versus the reference lozenge (Table 3). Each prototype lozenge and the reference lozenge achieved C_max_ at a median of 1.5 hours (Table 2). 

### Safety 

A total of 39 subjects received at least one of the four treatments, including 38 subjects who received prototype lozenge I, 37 who received prototype lozenge II, 39 who received prototype lozenge III, and 38 who received the reference lozenge. No AEs, SAEs, or deaths were reported during this study, and no laboratory or clinical abnormalities were reported. 

## Discussion 

Using the ratio of two nicotine metabolites, *trans*-3’-hydroxycotinine (3HC) and cotinine (COT), previous studies have demonstrated racial and ethnic differences in nicotine metabolism, including a significantly lower rate of nicotine metabolism in Asian subjects compared with Whites [12]. The rate of nicotine metabolism and clearance is largely determined by the cytochrome P450 (CYP) 2A6 enzyme [13], and a study that detailed the frequency of *CYP2A6* polymorphisms in a South Indian population found that CYP2A6*2 (rs1801272) genotype and allele frequencies were significantly lower in South Indians compared to Caucasians, Finns, and Spaniards, while the genotype and allele frequency of CYP2A6*4A (gene deletion) was significantly higher in South Indians compared with Chinese subjects and significantly lower in comparison to Caucasians, African Americans, Finns, and Spaniards. Despite differences in nicotine metabolism, however, observational data from a racially and ethnically diverse population suggested that non-White smokers are as likely to benefit from NRT as White smokers [15]. Additional studies are needed to characterize the precise relationship between nicotine metabolism and the efficacy of NRT in Asian and Indian populations. 

This study achieved the objective of comparing the PK parameters of three prototype 4-mg nicotine lozenges produced in India to a globally marketed reference 4-mg nicotine lozenge. The PK data demonstrated the bioequivalence of all three prototypes with the reference lozenge. As these three bioequivalent prototypes differ in their in vitro dissolution properties and the rate of nicotine release, these results suggest that the performance from the in-vitro method deployed is not a firm predictor of PK behavior of the nicotine molecule, and that dissolution specifications for nicotine lozenges can be widened to include the range tested in this study. 

## Conclusion 

The results of this study demonstrated the bioequivalence of all three prototype 4-mg nicotine lozenges to the globally marketed 4-mg nicotine lozenge based on the PK parameters of AUC_0–t_ and C_max_. Furthermore, all three prototypes were well tolerated, producing no AEs or medical abnormalities among subjects. These results support the use of locally produced nicotine lozenges, potentially increasing the access to NRT among inhabitants of India and helping to combat smoking rates in the country. These data add important information on the PK profiles of nicotine lozenges in the Indian population. 

## Funding 

This study was sponsored by GlaxoSmithKline Consumer Healthcare. Medical writing assistance was provided by Peloton Advantage and was funded by GlaxoSmithKline Consumer Healthcare. 

## Conflict of interest 

Reena Srivastava is an employee of GlaxoSmithKline Consumer Healthcare. 

Aditya Kaushik and Manpreet Sukhija are former employees of GlaxoSmithKline Consumer Healthcare and were employed by GlaxoSmithKline Consumer Healthcare at the time this study was conducted. 

**Table 1.** Subject demographics and baseline characteristics (safety population).

Parameter	Subjects (N = 39)
Sex, n (%)	
Male	39 (100)
Female	0
Race, n (%)	
Asian	39 (100)
White	0
Black or African American	0
Age, mean (SD), y	28.7 (6.4)
Height, mean (SD), cm	165.8 (5.4)
Weight, mean (SD), kg	59.2 (6.2)
BMI, mean (SD), kg/m^2^	21.6 (2.1)
Average number of cigarettes smoked per day, mean (SD)	5.3 (1.9)
Approximate time after awakening for the first cigarette, mean (SD), min	17.8 (7.5)
Years of smoking, mean (SD)	3.3 (2.2)

BMI = body mass index; SD = standard deviation.

**Table 2.** Baseline-adjusted nicotine plasma PK variables.

Parameter	Prototype I (N = 38)	Prototype II (N = 37)	Prototype III (N = 39)	Reference (N = 38)
C_max_, mean (SD), (ng/mL)	18.18 (5.58)	18.11 (5.11)	17.11 (4.70)	18.67 (5.98)
t_max_, median (min, max), h	1.50 (0.50, 4.00)	1.50 (0.25, 6.00)	1.50 (0.75, 6.00)	1.50 (0.25, 3.00)
AUC_0–t_, mean (SD), ng×h/mL	87.13 (44.25)	85.69 (42.80)	84.59 (35.58)	90.03 (44.94)
AUC_0–∞_, mean (SD), ng×h/mL	98.14 (59.53)	97.01 (61.93)	95.45 (51.82)	102.44 (64.59)
T_1/2_, median (min, max), h	2.74 (1.72, 5.20)	2.77 (1.72, 6.14)	2.66 (0.81, 7.67)	2.71 (1.77, 6.25)
K_el_, median (min, max), L/h	0.25 (0.13, 0.40)	0.25 (0.11, 0.40)	0.26 (0.09, 0.85)	0.26 (0.11, 0.39)

AUC_0-t_ = area under the plasma concentration-vs.-time curve from zero extrapolated to the time of the last quantifiable sample; AUC_0-∞_ = area under the plasma concentration-vs.-time curve from zero extrapolated to infinity; C_max_ = maximum plasma nicotine concentration; K_el_ = plasma elimination rate constant; PK = pharmacokinetc; SD = standard deviation; T_1/2_ = plasma half-life; t_max_ = time to maximum plasma nicotine concentration.

**Table 3.** Statistical analysis of baseline-adjusted nicotine plasma PK variables.

Parameter	Prototype I (N = 38)	Prototype II (N = 37)	Prototype III (N = 39)	Reference (N = 38)
C_max_, ng/mL				
Geometric mean^a^	17.52	17.72	16.50	17.98
Ratio (prototype/reference)	0.974	0.985	0.918	
90% CI for ratio	0.929, 1.021	0.940, 1.033	0.876, 0.962	
AUC_0–t_, ng×h/mL				
Geometric mean^a^	80.33	80.52	78.24	83.63
Ratio (prototype/reference)	0.961	0.963	0.936	
90% CI for ratio	0.931, 0.991	0.933, 0.994	0.907, 0.965	
AUC_0–∞_, ng×h/mL				
Geometric mean^a^	88.48	88.51	86.06	92.53
Ratio (prototype/reference)	0.956	0.957	0.930	
90% CI for ratio	0.927, 0.987	0.927, 0.987	0.901, 0.960	
t_max_, h				
Median difference^b,c^	0.00	0.00	0.00	
95% CI^b,c^	0.00, 0.50	0.00, 0.25	0.00, 0.50	
p-value^c^	0.2208	0.5695	0.0063	

^a^Geometric means are exponentiated least-squares means of log-transformed variables. ^b^Based on Hodges Lehmann estimates. ^c^Based on Wilcoxon signed-rank test. AUC_0–t_ = area under the plasma concentration-vs.-time curve from zero extrapolated to the time of the last quantifiable sample; AUC_0–∞_ = area under the plasma concentration-vs.-time curve from zero extrapolated to infinity; CI = confidence interval; C_max_ = maximum plasma nicotine concentration; PK = pharmacokinetik; t_max_ = time to maximum plasma nicotine concentration.
